# Differential Roles of MAPK Kinases MKK3 and MKK6 in Osteoclastogenesis and Bone Loss

**DOI:** 10.1371/journal.pone.0084818

**Published:** 2014-01-06

**Authors:** David L. Boyle, Deepa Hammaker, Meghan Edgar, Mario M. Zaiss, Stefan Teufel, Jean Pierre David, Georg Schett, Gary S. Firestein

**Affiliations:** 1 Medicine, University of California San Diego, La Jolla, California, United States of America; 2 Global Health Institute, École Polytechnique Fédèrale de Lausanne, Switzerland; 3 Institute of Osteology and Biomechanics, University Medical Center Hamburg-Eppendorf, Hamburg, Germany; 4 Department of Internal Medicine 3, University of Erlangen-Nuremberg, Friedrich Alexander University, Erlangen, Germany; Faculté de médecine de Nantes, France

## Abstract

Bone mass is maintained by osteoclasts that resorb bone and osteoblasts that promote matrix deposition and mineralization. Bone homeostasis is altered in chronic inflammation as well as in post-menopausal loss of estrogen, which favors osteoclast activity that leads to osteoporosis. The MAPK p38α is a key regulator of bone loss and p38 inhibitors preserve bone mass by inhibiting osteoclastogenesis. p38 function is regulated by two upstream MAPK kinases, namely MKK3 and MKK6. The goal of this study was to assess the effect of MKK3- or MKK6-deficiency on osteoclastogenesis in vitro and on bone loss in ovariectomy-induced osteoporosis in mice. We demonstrated that MKK3 but not MKK6, regulates osteoclast differentiation from bone marrow cells in vitro. Expression of NFATc1, a master transcription factor in osteoclastogenesis, is decreased in cells lacking MKK3 but not MKK6. Expression of osteoclast-specific genes Cathepsin K, osteoclast-associated receptor and MMP9, was inhibited in MKK3−/− cells. The effect of MKK-deficiency on ovariectomy-induced bone loss was then evaluated in female WT, MKK3−/− and MKK6−/− mice by micro-CT analysis. Bone loss was partially inhibited in MKK3−/− as well as MKK6−/− mice, despite normal osteoclastogenesis in MKK6−/− cells. This correlated with the lower osteoclast numbers in the MKK-deficient ovariectomized mice. These studies suggest that MKK3 and MKK6 differentially regulate bone loss due to estrogen withdrawal. MKK3 directly mediates osteoclastogenesis while MKK6 likely contributes to pro-inflammatory cytokine production that promotes osteoclast formation.

## Introduction

Homeostatic maintenance of bone mass and strength requires the concerted actions of bone-forming osteoblasts and bone-resorbing osteoclasts [1]. Imbalances in bone remodeling that favor osteoclast differentiation, survival and activation occur in chronic inflammatory diseases such as rheumatoid arthritis (RA) as well as in post-menopausal osteoporosis [2], [3]. Pro-inflammatory cytokines such as IL-1, TNF, and IL-6 in chronic inflammation or the loss of estrogen in menopause regulate bone resorption by increasing the expression of Receptor Activator of NF-κB ligand (RANKL) on osteoblasts and stromal cells or by amplifying RANKL-mediated differentiation of monocytes to osteoclasts [4]–[7]. The interaction of RANKL with its cognate receptor RANK, expressed on osteoclasts, initiates signaling that enhances osteoclast activity, leading to accelerated bone loss and fractures [8].

RANK and RANKL belong to the TNF receptor superfamily [9], [10]. The binding of RANKL to RANK recruits the adapter protein TNF receptor associated factor-6 (TRAF6) to the plasma membrane [11]. RANK, RANKL, and TRAF6 are essential for osteoclastogenesis as mice lacking these molecules have profound defects in bone resorption [11]. The RANK/TRAF6 complex activates several pathways including NF-κB and the MAPKs, JNK and p38, which induce the expression of NFATc1, considered one of the master transcription factors of osteoclastogenesis [12], [13]. NFATc1, in conjunction with the transcription factors AP-1, PU.1 and micropthalmia transcription factor (MITF), is required for the expression of osteoclast specific genes such as tartrate-resistant acid phosphatase (TRAP) and Cathepsin K that promote bone resorption and demineralization [14], [15].

The p38 family of MAPKs, most notably p38α, is an essential regulator of RANKL-mediated osteoclastogenesis, which makes it a potential therapeutic target for osteoporosis [16]. Several studies have shown that p38 inhibitors prevented inflammatory bone destruction in animal models and in some cases, reversed the existing disease [17]–[19]. MAPK kinases, MKK3 and MKK6, are two upstream enzymes that differentially regulate p38 function [20]. Our previous studies have shown that MKK3 is required for optimal p38α activation while MKK6 regulates pro-inflammatory cytokine production in response to LPS and TNF [21]. In addition, either MKK3- or MKK6-deficiency reduces arthritis severity and joint destruction in passive serum transfer K/BxN arthritis or collagen-induced arthritis [21]–[23]. The goal of this study was to assess the role of MKK3 and MKK6 in p38-regulated bone resorption. Therefore, we evaluated the effect of MKK3- and MKK6-deficiency on osteoclastogenesis in vitro and on bone preservation in vivo using an ovariectomy model of osteoporosis. These studies show that blocking either MKK3 or MKK6 has beneficial effects on bone preservation by differentially regulating osteoclastogenesis or function.

## Materials and Methods

### Mice

Female wildtype (WT) C57Bl/6 mice (6–8 weeks old) were purchased from Charles River Laboratories (Wilmington, MA). Female MKK3−/− and MKK6−/− mice (C57Bl/6 background) were obtained from Dr. Richard Flavell (Yale University, CT).

### Ethics Statement

All experimental animal protocols were reviewed and approved by the University of California San Diego Institutional Animal Care and Use Committee. This study was carried out in strict accordance with the recommendations in the Guide for the Care and Use of Laboratory Animals of the National Institutes of Health. All surgeries were performed under isoflurane vaporizer, and all efforts were made to minimize suffering. Mice were housed using standard housing conditions and allowed access to food and water ad libitum.

### Generation of Osteoclasts

Bone marrow was isolated from WT, MKK3−/−, and MKK6−/− femora and tibias. The red blood cells were lysed and the remaining cells were counted and seeded in T75 flasks at 1×10^6^ cells/ml. The cells were cultured for 24 h in αMEM (1X) containing GlutaMAX (Invitrogen, Carlsbad, CA) supplemented with 10% FBS, 5% penicillin-streptomycin, and 30 ng/ml of recombinant murine M-CSF (Peprotech, Rocky Hill, NJ). After 24 hours, the non-adherent cells were seeded in 24-well plates (1×10^6^ cells/ml) with 30 ng/ml of M-CSF and 50 ng/ml of receptor activator of NF-κB ligand (RANKL) (R&D, Minneapolis, MN). The culture medium was replaced every 72 h and supplemented with M-CSF and RANKL for 5–8 days until multinucleated osteoclasts were observed. In some experiments, cells were cultured for 5 days with 3 µM SB203580 (p38 inhibitor) or DMSO (EMD Chemicals, Gibbstown, NJ).

### Osteoclast Differentiation and Activity

Tartrate-resistant acid phosphatase (TRAP) staining: Differentiated cells were stained using the Acid Phosphatase, Leukocyte (TRAP) kit (Sigma-Aldrich, St. Louis, MO). Osteoclasts were defined as multinucleated (≥3 nuclei), TRAP positive cells and were counted in 5 pre-defined locations in each well. The data are represented as average count in each well.

Bone resorption assay: Bone marrow monocytes (BMM) were seeded at 3×10^5^ cells/well for 8 days on an Osteologic™ Bone Cell Culture System (BD Biocoat™, Bedford, MA) (4). The cell culture system was then subjected to von Kossa staining. Eight defined areas of each well were imaged and the resorption pits were quantified using Image Pro Plus software (Media Cybernetics, Bethesda, MD). The average resorption was calculated per area for each well and the data are expressed as a percentage of resorbed area/total area.

### Ovariectomy

At 16 weeks of age, female WT, MKK3−/−, and MKK6−/− mice were subjected to ovariectomy or sham-surgery and allowed to recover for 6 weeks. To confirm ovariectomy, leutinizing hormone (LH) was measured in serum by sandwich ELISA (Ligand assay and analysis core lab at the University of Virginia).

### Micro-Computed Tomography (micro-CT) and Bone Histomorphometry

Micro-CT images of the tibias were acquired as described previously [13]. Briefly, the tibias were harvested, fixed in 10% neutral buffered formalin for 24 h and stored in 70% ethanol. All quantifications were performed with digital image analysis (OsteoMeasure; Osteometrics, Atlanta, GA). The following parameters were measured: fraction of bone volume of the total sample volume (bone volume/tissue volume (BV/TV)), trabecular number (Tb.N), trabecular thickness (Tb.Th), and trabecular separation (Tb.Sp).

Histomorphometric analysis was performed on methacrylate-embedded undecalcified plastic sections of bone and were stained using von Kossa’s stain and TRAP stain. To measure bone formation rate (BFR), all mice were injected with calcein green solution (30 mg/kg, Sigma, MO) at 10 days and 2 days prior to harvesting. The tibias were prepared as described above and the measurements were performed using an image analysis system (OsteoMeasure) and the bone formation rate was calculated and the data are shown as µm^3^/µm^2^/year.

### Gene Expression Analysis

Total RNA was isolated from cells and 500 ng RNA was used for first-strand cDNA synthesis (Life Technologies, NY). Quantitative real-time PCR was performed using Taqman gene expression assay primer/probe sets and GeneAmp 7300 system (Applied Biosystems, Foster City, CA) [24]. Ct values were normalized to hypoxanthine-guanine phosphoribosyl transferase 1 (Hprt1) using the ΔΔCt method.

### Western Blot Analysis

Osteoclasts were lysed using a lysis buffer containing 50 mM HEPES (pH 7.4), 150 mM NaCl, 25 mM MgCl_2_, 1 mM EDTA (pH8.0), 10% glycerol, 1% Triton X-100, 20 mM ß-glycerophosphate, 10 mM NaF, 1 mM Na_2_VO_4_, 0.4% cocktail protease inhibitor (Roche, Indianapolis, IN). Protein was quantified using a Micro BCA protein assay kit (Thermo Scientific, Waltham, MA) and separated using a Nupage® Bis-Tris 4–12% polyacrylamide gel (Invitrogen) and transferred onto a PVDF membrane. The membrane was probed with antibodies specific for mouse phospho-p38 (Sigma), total p38 (Cell Signaling Technology, Danvers, MA), and Actin (Sigma, MO). Western blots were developed using the Immun-star™ Western C Kit and images were analyzed using the Versadoc Imaging System and Quantity One software (Bio-Rad Laboratories, Hercules, CA).

### Statistical Analysis

Comparisons between WT, MKK3−/−, and MKK6−/− cells were analyzed by one-way ANOVA and Tukey’s or Kruskal-Wallis multiple comparison tests, unless otherwise stated. Ovariectomy data were analyzed by two-way ANOVA and Tukey’s multiple comparison test. Data were analyzed using GraphPad Prism 6.0 and the comparisons were considered statistically significant if *p*<0.05.

## Results

### Effect of MKK3- and MKK6-deficiency on Osteoclastogenesis in vitro

**Figure 1:**
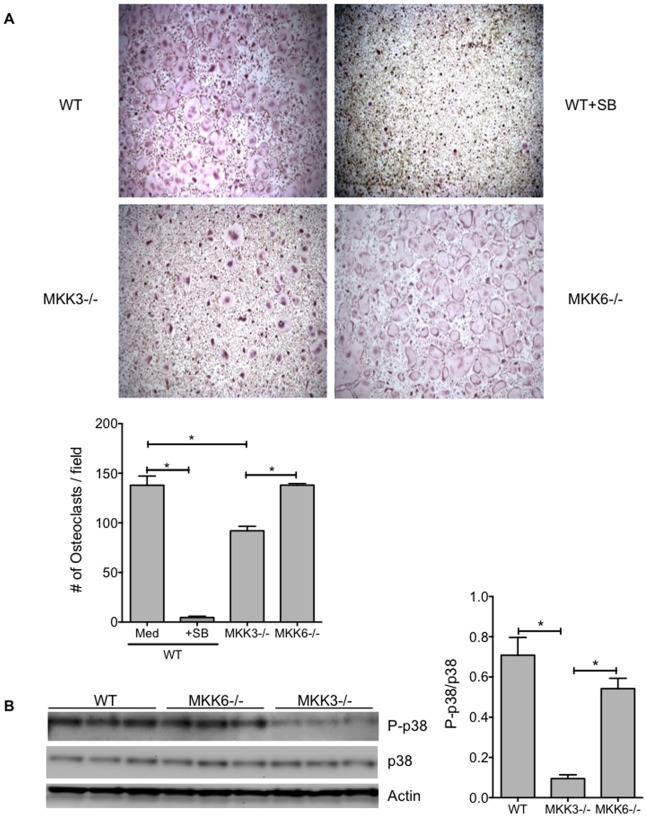
Effect of MKK-deficiency in osteoclastogenesis in vitro. (A) WT, MKK3−/− and MKK6−/− bone marrow cells were cultured to induce osteoclastogenesis and analyzed by TRAP staining (n = 3/group). The number of osteoclasts were counted in 5 pre-defined locations per well and the data are represented as mean ± SEM. Numbers of osteoclasts were reduced in SB-treated WT and MKK3−/− cells compared with WT. Osteoclastogenesis in MKK6−/− group were comparable to WT cells. *p<0.05 (B) Phosphorylation of p38 in MKK-deficient osteoclasts. Western blot analysis showed that P-p38 levels were significantly lower in MKK3−/− but not in MKK6−/− compared with WT group. *p<0.05.

To evaluate the role of MKK3 and MKK6 in osteoclast differentiation, we cultured WT and MKK3- and MKK6- deficient bone marrow cells in the presence of M-CSF and RANKL. WT cells were cultured in the presence or absence of the p38 inhibitor SB203580 (SB). Osteoclastogenesis was significantly decreased in MKK3−/− (p = 0.0012) and SB-treated WT group (p<0.0001) compared with WT control (Figure 1A, n = 3 mice/group). MKK3−/− osteoclast numbers were also significantly lower than MKK6−/− cells (p = 0.0012). Surprisingly, osteoclast numbers in MKK6−/− group were comparable to the WT control. We next determined the level of phosphorylation of p38 (P-p38) in cultured WT, MKK3−/− and MKK6−/− osteoclasts by Western blot analysis. MKK3−/− cells had significantly reduced P-p38 levels compared with WT or MKK6−/− (Figure 1B, p = 0.0009 and p = 0.005, respectively). These data show that MKK3, but not MKK6, is an important regulator of osteoclast differentiation in vitro.

### Bone Resorption Activity in MKK3- and MKK6-deficient Osteoclasts

**Figure 2:**
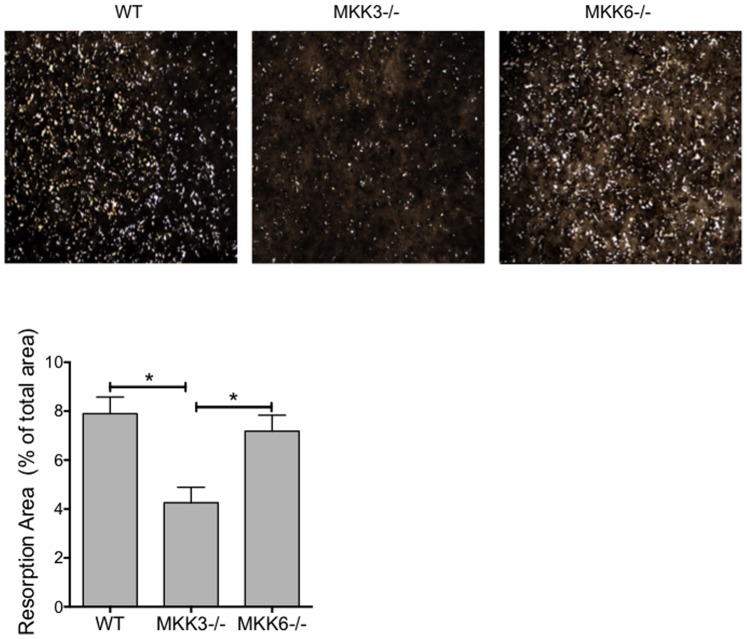
Bone resorption by MKK3- and MKK6-deficient osteoclasts. WT, MKK3−/− and MKK6−/− bone marrow cells were differentiated into osteoclasts on a calcium matrix and subjected to Von Kossa staining to determine resorption activity. The resorption pit area was calculated in eight areas/well and data are shown as percent resorbed area/total area. Resorption activity was significantly reduced in MKK3−/− but not MKK6−/− osteoclasts compared with WT. n = 3/group, *p<0.05.

Bone resorption of MKK-deficient osteoclasts was then determined by their ability to demineralize matrix and form resorption pits. Resorption was visualized using Von Kossa staining (Figure 2). MKK3−/− osteoclasts had a significantly lower number of resorptive pits than WT (p = 0.0061) or MKK6−/− cells (p = 0.027, Kruskal-Wallis test), suggesting that MKK3 is a key regulator of osteoclast differentiation in vitro.

### Gene Expression in MKK3−/− and MKK6−/− Osteoclasts

**Figure 3:**
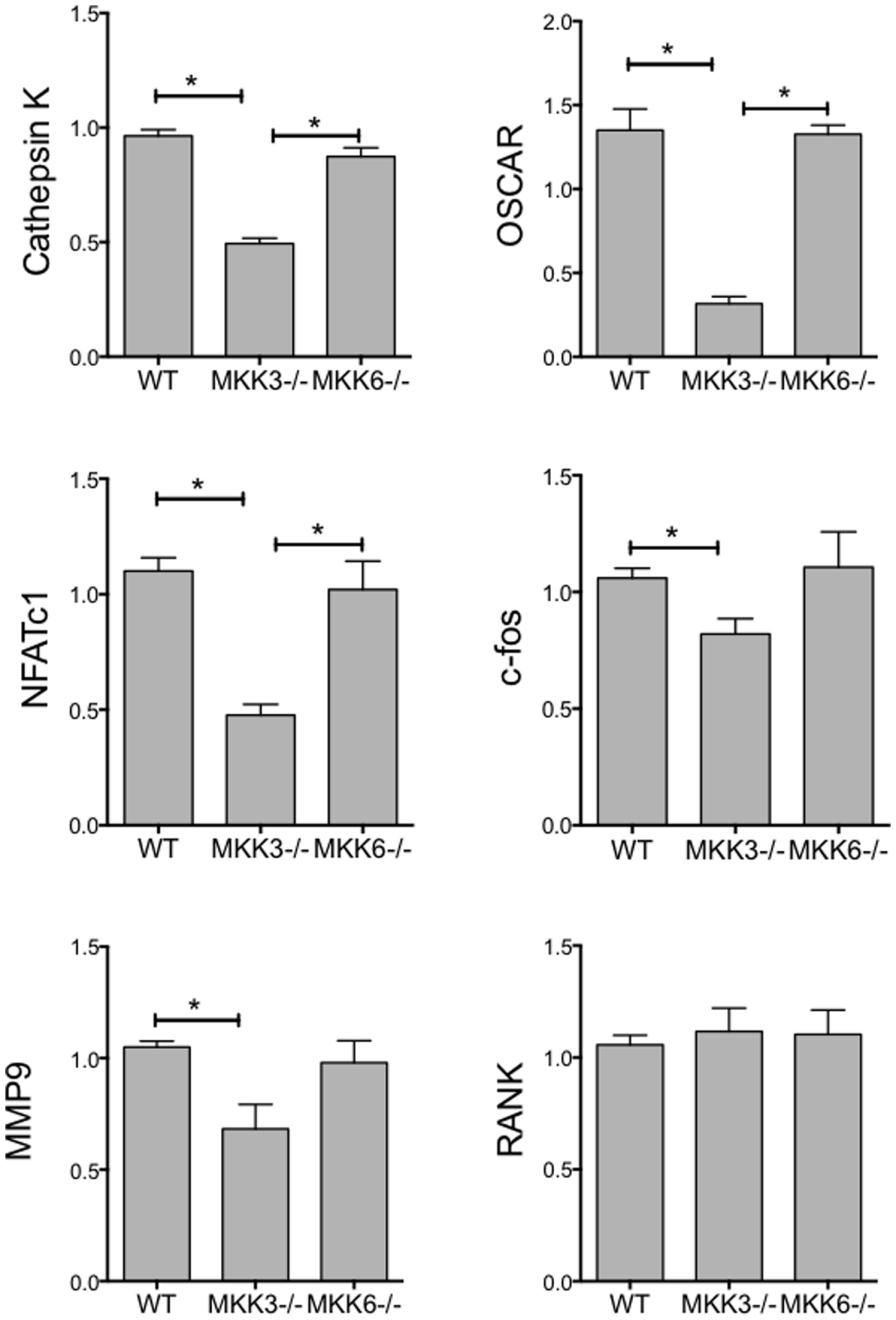
Gene expression in MKK3−/− and MKK6−/− osteoclasts. WT, MKK3−/− and MKK6−/− osteoclast RNA was isolated and subjected to qPCR using gene-specific primer probe sets (n = 3/group). Expression of NFATc1, cFos, Cathepsin K, OSCAR and MMP9 was significantly lower in MKK3−/− but not MKK6−/− cells, compared with WT. RANK expression was comparable in WT, MKK3−/− and MKK6−/− groups. *p<0.05.

Osteoclast differentiation requires the expression of a number of well-characterized markers such as NFATc1 and Cathepsin K, which regulate osteoclast function. To determine the effect of MKK-deficiency on these markers, we compared gene expression in WT, MKK3−/−, and MKK6−/− osteoclasts by qPCR (Figure 3). Expression of Cathepsin K and osteoclast-associated receptor (OSCAR) was significantly reduced in MKK3−/− osteoclasts compared with WT (p<0.001, respectively) or MKK6−/− cells (p<0.001, respectively). Similarly, NFATc1 expression was significantly reduced in MKK3−/− cells compared with WT (p = 0.004) or MKK6−/− osteoclasts (p = 0.009). Expression of c-fos and MMP9 (gelatinase B) was inhibited only in MKK3−/− cells (p = 0.037 and p = 0.031, respectively, t-test) compared with WT. In contrast, RANK expression was not decreased in MKK-deficient cells. These data show that in vitro expression of osteoclast-specific markers is inhibited in the absence of MKK3.

### Ovariectomy-induced Bone Loss in MKK3- and MKK6-deficient Mice

**Figure 4:**
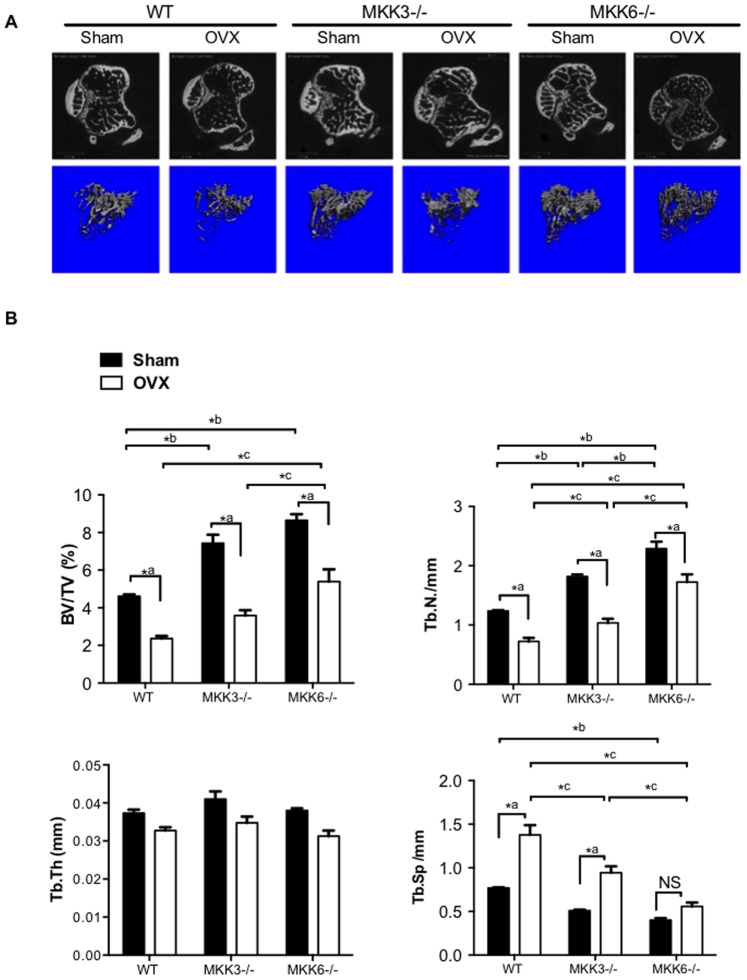
Ovariectomy-induced bone loss in MKK3−/− and MKK6−/− mice. (A) WT, MKK3−/− and MKK6−/− mice were subjected to ovariectomy and bone loss was calculated using micro-CT analysis (n = 5/group). Representative images are shown. Significant bone loss was observed in all three groups. (B) Bone volume and trabecular number were elevated in MKK3−/− and MKK6−/− sham-treated or OVX mice compared with respective WT controls. Trabecular thickness was similar in sham-treated and OVX mice. Trabecular separation was significantly higher in WT and MKK3−/− mice after OVX. MKK3- and MKK6-deficient mice had high bone mass phenotype compared with WT in sham-treated or OVX groups.

Loss of estrogen in ovariectomized (OVX) mice is associated with increased bone turnover with bone resorption exceeding formation, leading to a net bone loss. Thus far, our data have shown that MKK3-deficiency decreases osteoclastogenesis and bone resorption in vitro, suggesting that a lack of MKK3 might preserve bone in vivo. We therefore examined the effect of MKK-deficiency on bone preservation after ovariectomy. The efficiency of the surgery was confirmed by showing increased levels of luteinizing hormone (LH) in post-surgical sera (Figure S1). Trabecular bone density at the proximal tibia was evaluated in OVX or sham control mice using micro-CT (Figure 4A, n = 5/group).

Micro-CT analysis showed significantly higher bone volume (BV/TV) and trabecular number (Tb.N.) in sham MKK3−/− mice compared with WT sham control (Figure 4B, BV: p = 0.003, Tb.N: p = 0.006). Surprisingly, sham MKK6−/− mice had a significantly elevated bone volume and Tb.N. compared with sham WT control (BV: p<0.0001, Tb.N: p<0.0001). Together, these data suggest that both MKK3 and MKK6 play a role in physiological bone homeostasis. While a significant bone loss was observed after OVX in all three genotypes, bone volume and Tb.N were modestly elevated in OVX MKK3−/− and significantly increased in MKK6−/− mice compared to the OVX WT control. These results suggest that MKK6−/− mice were partially protected from OVX-mediated bone loss, maintaining a bone volume similar to sham-treated WT controls. Trabecular thickness was virtually unchanged in OVX or sham control WT and MKK−/− mice. In contrast, trabecular separation (Tb. Sp) was significantly higher after OVX in WT (p = 0.002) and MKK3−/− (p = 0.005) but not in MKK6−/−, compared with their respective sham controls. Trabecular separation was significantly lower in OVX MKK3−/− (p = 0.003) and MKK6−/− (p<0.0001) compared with OVX WT mice, indicating a higher bone mass in MKK-deficient mice. Together, the in vivo data demonstrate that MKK3- and MKK6-deficiency are associated with a high bone mass phenotype, which is partially maintained after OVX.

### Histomorphometric Analysis of Bone in Ovariectomized MKK3- and MKK6-deficient Mice

**Figure 5:**
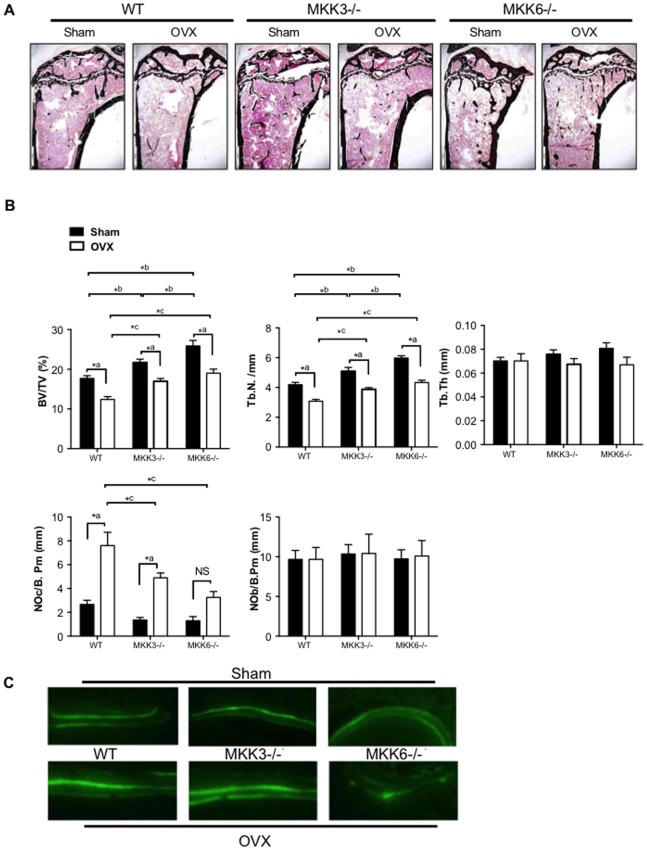
Histomorphometric analysis of bone in ovariectomized MKK3−/− and MKK6−/− mice. (A) Tibial bone sections from sham and OVX-treated mice were stained and representative figures are shown (n = 5/group) (B) Histomorphometric analysis showed that bone volume and trabecular number were elevated in MKK6−/− mice compared with WT after sham surgery or OVX. Trabecular thickness was unaltered after OVX in the three groups. TRAP-staining showed lower osteoclast numbers (NOc/B.Pm) after OVX in MKK3−/− and MKK6−/− compared with WT mice. Osteoblast numbers were unaltered after OVX in the three groups. (C) Bone formation rate was evaluated by injecting mice with calcein green solution at 10 days and 2 days prior to harvesting. The tibias were evaluated for bone formation rate (n = 3/group). Bone formation rate was unaltered by MKK-deficiency in sham- or OVX-treated mice compared with WT controls. *p<0.05.

To further evaluate the effect of MKK3- and MKK6-deficiency in estrogen-deficient mice, undecalcified tibial bone sections from OVX and sham control mice were stained and subjected to histomorphometric analysis (Figure 5A, B). Consistent with the micro-CT data, bone volume (%BV/TV) was significantly higher in sham- and OVX-treated MKK3−/− (p<0.05 for each) and MKK6−/− mice compared to respective WT controls (p<0.001, for each). OVX led to a reduction of bone volume in the WT, MKK3−/− and MKK6−/− groups (n = 5/treatment, p<0.001, respectively). Nonetheless, bone volume and trabecular number were at least partially maintained in MKK-mutants, indicating a similar bone volume after OVX as sham-treated WT mice. Trabecular thickness (Tb.Th) was similar in WT, MKK3−/−, and MKK6−/− mice, both after sham-treatment and OVX.

TRAP staining showed higher osteoclast numbers (NOc/bone perimeter) in OVX WT mice compared with sham-treated WT mice (p<0.001). However, MKK3- and MKK6-deficient mice had significantly lower osteoclast numbers compared with sham-treated or OVX WT mice (MKK3−/−: p<0.01, MKK6−/−:p<0.001). Interestingly, the number of osteoblasts (NOb) and bone formation rate was not altered in MKK3 and MKK6 mutants. These results suggest that MKK-deficiency inhibits osteoclast formation in vivo but does not alter numbers of osteoblasts or bone formation (Figure 5C). Together, these data show that MKK-deficiency induced high bone mass by lowering osteoclast numbers in vivo.

## Discussion

Osteoclast differentiation and activation is regulated by p38 MAPK, at least in part by mediating the effects of pro-inflammatory cytokines [25], [26]. Several studies have shown that p38 inhibitors protect against bone resorption in inflammation, possibly through the direct effects on osteoclasts as well as through inhibition of p38-induced cytokine expression. For instance, p38α activation is essential for TNF-mediated inflammatory bone loss, and a selective p38α inhibitor prevented trabecular bone loss and increased cortical bone area in ovariectomized rats [13], [27]. Our previous studies demonstrated clinical benefits of MKK3- and MKK6-deficiency in acute and chronic models of arthritis [21]–[23]. Inflammation-induced bone loss in the joints was markedly reduced in the absence of MKK3 or MKK6, with a corresponding decrease in serum IL-6 and synovial MMP [22]. In the present study, we extend our observations on bone protection by evaluating the effect of MKK3- and MKK6-deficiency on osteoclastogenesis and bone loss due to estrogen withdrawal in ovariectomized mice.

Our data show that MKK3 and MKK6 differentially regulate osteoclastogenesis. MKK3 is required for optimal osteoclastogenesis and function. MKK6 was unable to effectively compensate for the absence of MKK3 in the osteoclasts. MKK6-deficiency, on the other hand, did not affect osteoclastogenesis in vitro. Consistent with our previous studies, P-p38 levels were markedly reduced in MKK3−/− but normal in MKK6−/− osteoclasts [21]. Osteoclast numbers and resorption activity correlated with p38 activation in MKK3−/− and MKK6−/− cells and are in agreement with previous studies [16]. Expression of NFATc1 and c-fos were reduced in MKK3−/− osteoclasts, indicating that p38 function is necessary for the induction of these transcription factors that are involved in osteoclast differentiation [28]. Cathepsin K, OSCAR, and matrix metalloproteinase 9 (MMP9) expression was reduced in MKK3-deficient osteoclasts. This is consistent with the requirement of NFATc1 in the transcription of these osteoclast-specific markers [15], [29], [30]. Together, these results indicate that MKK3, rather than MKK6, is a principal regulator of RANKL-induced signaling in osteoclastogenesis in vitro. These data are in accordance with other studies showing that p38 regulates the early phases of osteoclast differentiation [31], [32]. However, those studies suggested that MKK6, but not MKK3, is necessary for osteoclastogenesis in vitro, where it enhances cell survival but not bone resorption. The differences might be partly due to differences in the methodology, such as the efficiency of inhibiting MKK3 and MKK6 using viral transduction of dominant-negative MKK3 and MKK6 constructs compared with using bone marrow-derived osteoclasts of MKK3−/− and MKK6−/− mice.

Osteoclastogenesis in vivo is regulated by a number of factors such as vitamin D, parathyroid hormone, prostaglandins and most notably, estrogen [33]–[36]. Estrogen is a key modulator of both the immune system and bone homeostasis [37]. It regulates bone remodeling by decreasing osteoclast life span by promoting apoptosis [38], [39]. Estrogen inhibits expression of RANKL on osteoblasts, stromal cells, and lymphocytes, and increases production of osteoprotegerin (OPG), an inhibitor of RANK signaling [1]. In menopause, the loss of estrogen is associated with elevated levels of IL-1, IL-6, TNF, and M-CSF, which support bone resorption [40], [41]. Estrogen deficiency also triggers the differentiation of Th17 cells that produce the pro-inflammatory cytokine IL-17 [42]. IL-17 induces osteoblast and stromal cell production of IL-6, and TNF, which induce RANKL expression, accelerate osteoclastogenesis and lead to osteoporosis [43].

In this study, we showed that both MKK3- and MKK6-deficiency is associated with high bone mass and at least partially inhibited bone loss after ovariectomy, even though our in vitro studies did not show major effects of MKK6 on in vitro osteoclastogenesis. Histomorphometric data demonstrated that high bone mass in MKK3 and MKK6-deficiency correlates with reduced osteoclast numbers. The decrease in osteoclast numbers in MKK6-deficient mice could be due to decreased p38-regulated cytokine production, such as IL-6, since a direct effect on osteoclasts was not observed. The bone protection is consistent with our previous studies showing reduced inflammation and joint destruction in MKK3−/− and MKK6−/− mice, where a direct effect on osteoclasts for MKK3 and an indirect effect by blocking cytokines for MKK6 were found [22].

The study also found no major effect on osteoblast number and bone formation rate in MKK3- and MKK6- deficient mice. The regulation of bone homeostasis and skeletal formation was recently evaluated in MKK3- and MKK6-deficient mice [44]. That study showed that both MKK3−/− and MKK6−/− mice had reduced bone mass resulting from defective osteoblast differentiation. The reason for the discrepancy is unclear but might be partly due to strain variations in mice and the different ages of the mice used, 5 weeks compared with 20 weeks for our study.

In conclusion, MKK3 and MKK6 affect bone mass through regulation of osteoclast numbers in vivo. MKK6 and to a lesser extent MKK3 mitigate bone loss in conjunction with estrogen deficiency. While MKK3 likely mediates direct effects on osteoclasts through RANKL signaling, MKK6 likely contributes to the production of pro-inflammatory cytokines such as IL-6 and IL-17 that increase RANKL expression. Targeting upstream p38 kinases, such as MKK3 and MKK6 might modulate bone loss during inflammation and estrogen deficiency.

## Supporting Information

Figure S1 **Serum leutinizing hormone (LH) in ovariectomized mice.** The efficiency of the ovariectomy was evaluated by measuring LH in sera. LH levels were significantly elevated in ovariectomized WT, MKK3−/−, and MKK6−/− sera compared with sham-treated sera (n = 8/group, *p<0.05).
(TIF)
