# Correction: Differential Roles of MAPK Kinases MKK3 and MKK6 in Osteoclastogenesis and Bone Loss

**DOI:** 10.1371/annotation/4fbfa4f0-cb27-4ec4-80d6-3b0ed5e9e8b8

**Published:** 2014-01-16

**Authors:** David L. Boyle, Deepa Hammaker, Meghan Edgar, Mario M. Zaiss, Stefan Teufel, Jean Pierre David, Georg Schett, Gary S. Firestein

There was an error in the Funding statement. The correct funding is:

"Supported by the NIH grants R01 AR047825 and R01 AI07055. The funders had no role in study design, data collection and analysis, decision to publish, or preparation of the manuscript."

